# α-Bisabolol alleviates doxorubicin-induced cognitive dysfunction in rats via enhancing the hippocampal BDNF/TrKB signaling and inhibiting neuroinflammation

**DOI:** 10.3389/fphar.2025.1549009

**Published:** 2025-03-07

**Authors:** Sara T. Elazab, Walter H. Hsu

**Affiliations:** ^1^ Department of Pharmacology, Faculty of Veterinary Medicine, Mansoura University, Mansoura, Egypt; ^2^ Department of Biomedical Sciences, College of Veterinary Medicine, Iowa State University, Ames, IA, United States

**Keywords:** chemobrain, cognitive impairment, doxorubicin, α-bisabolol, oxidative stress, BDNF/TrKB signaling

## Abstract

Chemofog is a serious sequela commonly manifested among cancer patients receiving doxorubicin (DOX) chemotherapy. Our goal was to explore the abrogative action of α-Bisabolol (BISA), a phytochemical sesquiterpene, against DOX-induced cognitive deficit. Rats were allocated into 5 groups: Group I: control; Group II received BISA orally (100 mg/kg/day for 4 weeks); Group III received DOX (2 mg/kg/week/i.p.) for 4 weeks; Groups IV and V were administered BISA orally at 50 and 100 mg/kg, respectively plus DOX, i. p. Results: 1) BISA attenuated DOX-induced chemofog as shown in memory-related behavioral tests. 2) BISA restored the hippocampal histological structure and redox homeostasis via diminishing MDA content and upregulating Nrf2 and HO-1 genes. 3) BISA mitigated DOX-induced neuroinflammation through reducing NF-kB, TNF-α, IL-6, IL-1β, and GFAP expressions. 4) BISA repressed the hippocampal apoptosis via downregulating Bax gene and upregulating Bcl-2 gene. 5) BISA enhanced the synaptic plasticity by activating the BDNF/TrKB signaling and increasing the levels of neurotransmitters that enhance memory, i.e., ACh, 5-HT, and DA. BISA at 100 mg/kg/day exerted a better neuroprotection than BISA at 50 mg/kg/day. Thus, BISA may protect cancer patients from cognitive disorders caused by DOX.

## Graphical Abstract

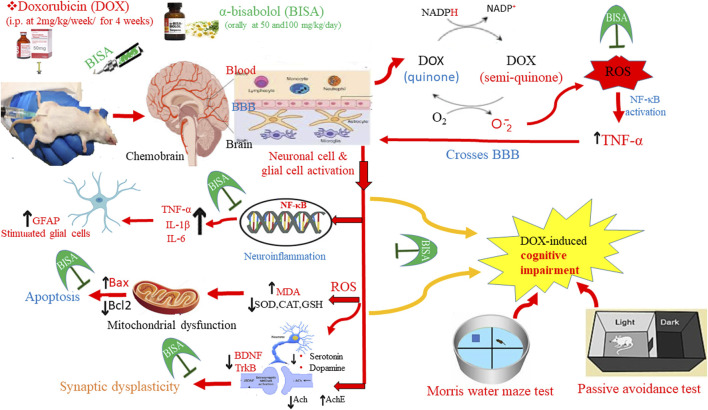

## 1 Introduction

Cancer chemotherapy is usually associated with a wide range of devastating side effects that seriously impair cancer survivors' quality of life (Ahles and Saykin, 2007). Chemotherapy-induced cognitive impairment (CICI), also known as chemobrain or chemofog, is the most detrimental adverse effect manifested by weakened memory, reduced learning and processing speed, and decreased attention either throughout or following chemotherapy (Koppelmans et al., 2012; Lange et al., 2019). Chemofog has been attributed to the tissue damage triggered by mostly the oxidative insult, neuroinflammation, and mitochondrial disruption; the exact underlying mechanism has yet to be fully understood. 75% of cancer patients suffer from this neurological disorder during chemotherapy (Flanigan et al., 2018; Shi et al., 2019). The majority of CICI cases have been reported among breast cancer-afflicted women undergoing chemotherapy (Jim et al., 2012).

Doxorubicin (DOX) is a well-known drug that is broadly utilized as a remedy for cancers of the breast, thyroid, prostate, ovary, and Hodgkin’s lymphoma. It is a member of the anthracycline group, which displays anticancer activity via 3 ways: interfering with DNA function, impacting the growth of cancer, blocking topoisomerase, releasing excessive free radicals (El-Agamy et al., 2019). These mechanisms destroy the essential biomolecules, thereby leading to cancer cell death. Unfortunately, the cytotoxic actions of DOX damage the healthy cells as well, causing toxicities of different organs such as cardiotoxicity, hepatotoxicity, nephrotoxicity, and neurotoxicity (Carvalho et al., 2009). Tangpong et al. (2006) and Ongnok et al. (2020) revealed that DOX can cause significant damages in the brain through a secondary mechanism by increasing peripheral cytokine tumor necrosis factor alpha (TNF-α) levels which penetrates the blood brain barrier (BBB) and impairs antioxidant defense, resulting in synaptic dysplasticity, death of neurons, and ultimately cognitive dysfunction.

Chemofog is associated with a reduction in neurotrophins concentrations which are necessary for neuron growth and survival (Saral et al., 2021). Brain-derived neurotrophic factor (BDNF) is one of the neurotrophins found in the brain with abundant expression in the hippocampus (Murer et al., 2001). It possesses neuroprotective properties as it participates in the regulation of glial cells and neurons proliferation and in the modulation of synaptic plasticity of both long- and short-term memories (Kowianski et al., 2018). Park et al. (2018) reported that the decrease in BDNF and its receptor, tropomyosin receptor kinase B (TrkB) accounted for the attrition of the recently formed neuronal cells in the hippocampus, resulting in the disturbance of working memory and spatial learning after administration of DOX. Currently, there is no effective and safe therapy for chemofog in cancer survivors. Numerous researches have highlighted the possible role of phytochemicals in ameliorating a variety of neurodegenerative conditions (Beking and Vieira, 2019; Maher, 2019). For instance, curcumin exhibited neuroprotective effect in parkinson’s disease model via upregulating Trk/phosphoinositide 3-kinases (PI3K) signaling and elevating BDNF levels (Yang et al., 2014). In addition, berberine has been proven to ameliorate cognitive disturbances caused by DOX via enhancing BDNF through activating SIRT-1 (Shaker et al., 2021).

Among several biologically active phytochemicals, Bisabolol (BISA), also called levomenol, has garnered considerable interest owing to its diverse pharmacological features, including anti-infective, anti-oxidant, anti-inflammatory, anti-nociceptive, gastroprotective, cardioprotective, and anti-cancer activities (Braga et al., 2009; Rocha et al., 2011; Forrer et al., 2013; Barreto et al., 2016; Meeran et al., 2020). It is a monocyclic sesquiterpene alcohol that was initially isolated from *Matricaria chamomilla* plant (Mckay and Blumberg, 2006). BISA is found in nature in two distinct forms, ά and β. The ά-isomer form is responsible for the majority of its biological characteristics. Owing to its high lipophilicity, BISA can easily undergo oxidation and generate two bisabolol oxides (A and B) (Arunachalam et al., 2022). Earlier studies have elucidated the palliative effect of BISA against cardiac and renal toxicity caused by DOX (Arunachalam et al., 2022; Meeran et al., 2023), cadmium-induced liver injury (Elazab and Hsu, 2022), neurodegeneration in rotenone (ROT)-provoked rat model of Parkinson’s disease (Javed et al., 2020), and neuronal injury and memory disorders caused by cerebral ischemia in mice (Fernandes et al., 2019). The neuroprotective effect of BISA against cognitive disturbances caused by DOX has not been explored. However, other sesquiterpenoids, e.g., β-caryophyllene have exhibited abrogative action against cognitive disorder to both long- and short-term memory caused by aspartame via enhancing the BDNF/TrKB signaling pathway (Rosa et al., 2023).

The present study was designed to assess the potential ameliorative action of BISA against DOX-induced cognitive dysfunction and the mechanisms implicated in this action. We hypothesize that BISA’s palliative effect on DOX-induced chemofog is through its antioxidant, anti-inflammatory, and anti-apoptotic properties, as well as its effect on the BDN/TrKB pathway.

## 2 Materials and methods

### 2.1 Chemicals

Doxorubicin injectable solution (2 mg doxorubicin hydrochloride/mL) was supplied from EBEWE Pharma Ges.m.b.H. Nfg. KG, Unterach am Attersee, Austria. α-Bisabolol (BISA, Cas No. 23089-26-1) was purchased from Sigma Aldrich Co. (St. Louis, MO, United States). BISA was prepared with sunflower oil (50 and 100 mg/mL for administration to rats at doses 50 and 100 mg/kg, respectively).

### 2.2 Animals

This research was carried out on 60 male Wistar rats (180–200 gm) obtained from the Laboratory Animal House (Zagazig University, Egypt). Rats were kept in plastic cages in well-controlled housing conditions with a temperature 24°C ± 1°C, humidity 60%–70%, and alternating shifts of 12 h dark and 12 h light. They were supplied with food pellets and drinking water *ad libitum* and were left to adapt to the experimental environment for 7 days before the commencement of the investigation. The procedures of this study were revised and authorized by the Mansoura University Animal Care and Use Committee.

### 2.3 Sample size calculation

Sample size was calculated depending on a former research by Egwuatu et al. (2023) employing G*power 3.1.9.4 software, according to the difference between two independent study groups (control Vs. DOX groups) utilizing t-test with a probability type I error (α) = 0.05 and a power 0.9. The sample size was estimated to be 30 rats (6 rats/group). A larger number of rats (12 rats/group) was enrolled in each experimental group at the beginning of the study as we expected that there will be mortalities in the groups receiving DOX and that some rats may be excluded in the training session of the passive avoidance test (those who failed to step into the dark chamber in the training session within 90 s).

### 2.4 Experimental design

The examined rats were randomly assigned to 5 groups (12 rats/group). Group I (control) was injected with normal saline (0.2 mL/rat, vehicle for DOX) intraperitoneally (i.p.) once/week at 0, 7, 14, 21 days of the trial and was administered sunflower oil (BISA vehicle) at 1 mL/kg/day orally for 4 weeks. Group II (BISA group) received normal saline i. p. once a week and was given BISA in sunflower oil by gavage at 100 mg/kg/day for 4 weeks. Group III (DOX group) was injected i. p. with DOX at 2 mg/kg/week (El-Agamy et al., 2018; Shaker et al., 2021) and was given sunflower oil orally every day for 4 weeks. Groups IV and V were administered DOX at 2 mg/kg i. p. weekly and BISA orally at 50 and 100 mg/kg/day, respectively, for 4 weeks. The doses for BISA were selected based on published reports (Javed et al., 2020; Fernandes et al., 2019; Cavalcante et al., 2020; Nazarinia et al., 2023).

### 2.5 Behavioral tests

#### 2.5.1 Passive avoidance test

A passive avoidance test was conducted to examine the short-term memory as illustrated by Abdel-Aziz et al. (2016) using the apparatus (Passive Avoidance Set-up, Ugo Basile, Italy). This device had two compartments, one was illuminated by a 10-W lamp, whereas the second chamber was dark with a grid floor that was set to shock the rats with an electric shock at a certain intensity when they passed on it. An automated sliding door connects these two chambers. The experiment was performed in two sessions for each rat; training and test one. In the training period (performed on the 28th day of the study), each rat was smoothly put in the lit compartment, and when the rat entered the black chamber, placing its feet on the floor, the door closed, and an electric shock (1 mA) was directed for 2 s for each animal. Rats that did not enter the dark chamber within 90 s were excluded from the study. One day after the training session, the test session was carried out (on the 29th day of the experiment). Rats were set in the lighted chamber, and the time taken to enter the dark chamber (known as the step-through latency) was monitored, and this time is regarded as a step-through reaction to assess the rat’s memory acquisition following the exposure to a painful stimulus. A cutoff period of 300 s was considered in the test, and rats were not subjected to electric shock in the dark chamber during the test session.

#### 2.5.2 Locomotion assessment

To exclude the influence of motor disorders on the step-through latency of passive avoidance test, the locomotor activity of rats was evaluated employing an animal activity detector (Opto-Varimax-Mini Model B; Columbus Instruments, OH, United States). Rat movements inside this device led to interruptions to infrared beams (scan rate = 160 Hz, wavelength = 875 nm, diameter = 0.32 cm, and spacing = 2.65 cm) that were counted. According to El-Agamy et al. (2018), rat’s locomotor activity was determined as counts/5 min.

#### 2.5.3 Morris water maze test (MWM) test

For assessing the learning ability and spatial memory, MWM test was performed as described by Jang et al. (2013). We used MWM apparatus with a circular pool (180 cm in diameter, 60 cm in height, containing water at 25°C–27°C). This pool was divided into 4 identical quadrants; one of them contains a concealed escape platform at 2 cm water depth. All animals were trained 3 times/day with platform position for 4 days. During each training trial, rats were allowed to swim from various release positions and trained to get into the concealed platform, where they were directed to remain there for 30 s prior to placing them in their cages. The time spent by each rat to find the platform (latency time) was registered. Rats that were unable to arrive at the platform within 90 s, were manually directed to it. After the last training session (on the fifth day of the test), animals were subjected to the probe trial test where the platform was eliminated and the rats were permitted to search for the eliminated platform for up to 90 s and the period spent by the animals in the platform quadrant was documented.

### 2.6 Samples collection

On the 32nd day of the experiment (at the end of behavioral tests after finishing MWM test), 6 rats from each group were cervically decapitated. Then, the entire brain from each rat was promptly excised, and the hippocampus was removed and divided into 3 portions. The first portion underwent homogenization process followed by centrifugation at 3,000*g* for 10 min, and the collected supernatant was utilized for investigating the redox status. The second portion was immersed in 10% formalin for the preparation of paraffin blocks required for microscopic examination of hippocampal tissues. The last portion was frozen at −80°C for gene expression assessment and enzyme-linked immunosorbent assay (ELISA).

### 2.7 Redox status in hippocampal tissues

By using spectrophotometer, the level of reduced glutathione (GSH), superoxide dismutase (SOD) and catalase activities (CAT) in the homogenate supernatant of hippocampal tissues were measured as described previously by Beutler (1963), Nishikimi et al. (1972), and Fossati et al. (1980), respectively. In addition, the technique of Ohakawa et al. (1979) was employed to evaluate malondialdehyde (MDA) content.

### 2.8 ELISA

ELISA was utilized to estimate the hippocampal levels of interleukin-1β (IL-1β), IL-6 (Bioassay Technology Laboratory Co. Shanghai, China), total Nuclear factor kappa B (NF-kB, the total protein level from both nuclear and cytoplasmic fractions), acetylcholinesterase activity (AChE), concentrations of Gamma-aminobutyric acid (GABA) (Fine Test Co., Boulder, CO, United States), acetylcholine (Ach, Elabscience Co. Houston, TX, United States), serotonin (5-HT, AFG Scientific, Wood Dale, IL, United States), dopamine (DA, Cusabio Co., Houston, TX, United States), glutamate (Novus Biologicals Co., Centennial, Co., United States), BDNF (Biosensis Pty Ltd., Thebarton, SA, Australia), and TrkB (Antibodies co. Cambridge, United Kingdom). The guidelines issued by the manufacturers were followed when using these ELISA kits.

### 2.9 Detection of the transcription levels of Nrf2, HO-1, Bax, and Bcl-2, P38 MAPK, and SIRT-1 genes in hippocampus by qRT-PCR

As per the directions recommended by the manufacturer, the QIAamp RNeasy Mini kit (Qiagen Inc., MD, United States) was implemented to isolate the total RNA from the hippocampal specimens of all experimental rats. The extracted RNA’s purity and concentration were assessed by a NanoDrop (UV-Vis spectrophotometer Q5000, United States). Afterwards, reverse transcription of RNA to cDNA was accomplished utilizing SensiFast™ cDNA synthesis kit (cat. No. Bio-65053, Bioline Ltd., United Kingdom). The mRNA levels of Nuclear factor erythroid 2-related factor (Nrf2), Heme oxygenase-1 (HO-1), Bax, and B-cell lymphoma 2 (Bcl-2), P38 mitogen-activated protein kinase (P38 MAPK), and Sirtuin-1 (SIRT-1) genes were evaluated with the aid of a Stratagene MX3005P real-time PCR machine (Agilent, CA, United States) employing SYBR Green PCR Master Mix (2x SensiFast™ SYBR, Bioline Ltd., United Kingdom). To normalize the expression of the examined genes, β-Actin, a reference housekeeping gene, was utilized. Table 1 displays the sequences of the used primers for the target genes. The qRT-PCR reaction was achieved under the following conditions: starting with initial denaturation at 94°C for 15 min (40 cycles), with subsequent heat activation for 15 s at 94°C, primers annealing at 68°C (30 s) for Bcl-2, 58°C (30 s) for P38 MAPK, and at 60°C (30 s) for the other remaining tested genes, and finally extension temperature was set at 72°C for 30 s. The 2^−ΔΔCT^ (Ct: cycle threshold) calculation was applied for relative estimation of the transcription levels of the analyzed genes (Yuan et al., 2006).

**TABLE 1:** The sequences of primers utilized for quantitative real-time PCR investigation.

Target gene	Forward primer (5′-3′)	Reverse primer (5′-3′)	References
Nrf2	CAC​ATC​CAG​ACA​GAC​ACC​AGT	CTA​CAA​ATG​GGA​ATG​TCT​CTG​C	Yamashita et al. (2014)
HO-1	GGC​TTT​AAG​CTG​GTG​ATG​GC	GGG​TTC​TGC​TTG​TTT​CGC​TC	Chu et al. (2020)
Bax	CACCAGCTCTGAACAGAT CATGA	TCAGCCCATCTTCTT CCAGATGGT	Kinouchi, (2003)
Bcl-2	CAC​CCC​TGG​CAT​CTT​CTC​CTT	AGC​GTC​TTC​AGA​GAC​AGC​CAG	
P38 MAPK	CGA​GCG​ATA​CCA​GAA​CCT​GT	GCG​TGA​ATG​ATG​GAC​TGA​AA	Wang et al. (2015)
SIRT-1	CAC​CAG​AAA​GAA​CTT​CAC​CAC​CAG	ACC​ATC​AAG​CCG​CCT​ACT​AAT​CTG	Santoro et al. (2021)
β-Actin	TCC​TCC​TGA​GCG​CAA​GTA​CTC​T	GCT​CAG​TAA​CAG​TCC​GCC​TAG​AA	Banni et al. (2010)

### 2.10 Microscopic examination of hippocampal samples

#### 2.10.1 Hematoxylin and eosin (H&E) staining

After preservation in 10% formalin, the hippocampal tissue samples of all groups were dehydrated with ascending grades of alcohol, cleared using xylene, and submerged in paraffin wax. Thereafter, a microtome was employed to slice 4 µm thick tissue sections, which were assembled on glass slides. The slides were scrutinized under a light microscope after staining with H&E as delineated by Suvarna et al. (2018). The degenerated neurons were quantified in CA3 and dentate gyrus from the slide of each rat for 6 rats/group. The grading and quantitative scoring of neuronal degeneration was conducted following the method of Khalil et al. (2022) [scale score: 0 = normal; 1 = mild (<25%, scattered neurons); 2 = moderate (25%–50%, scattered neurons); 3 = sever (>75%, scattered neurons); 4 = sever, multifocal]. The degenerated neurons were identified based on cell morphology, cytoplasmic appearance, nuclear condensation, and neurofibrillary integrity.

#### 2.10.2 Toluidine blue staining

Additional slides for the hippocampus were impregnated with 0.1% toluidine blue for 2 min. After dehydration of the sections, they were mounted on Canada balsam, and the slides were visualized by a microscope (Bancroft and Gamble, 2008). The neurons in the hippocampus were regarded as viable if they have round nuclei and observable nucleoli. In contrast, highly stained and shrunken cells indicate degenerated neurons which were counted in CA3 and dentate gyrus from every slide of each rat for 6 rats per experimental group. The number of damaged neurons was reported as a ratio out of 100 neurons in each area.

#### 2.10.3 Immunohistochemistry

TNF-α and glial fibrillary acid protein (GFAP) immunohistochemical staining in the hippocampal sections was carried out according to the description of Khafaga et al. (2021). Briefly, the deparaffinized sections were rehydrated utilizing ethanol at descending concentrations. After unmasking the antigen by boiling for 10–20 min at 105°C, the slides were rinsed with phosphate buffer saline (PBS). Then, the slides were dipped in 3% H_2_O _2_ (in pure methanol) for 5 min at 25°C to suppress the endogenous peroxidase. Thereafter, 10% normal goat serum was added and maintained for 1 h to abolish the non-specific reaction. The prepared hippocampal slides were subjected to 12 h incubation with the primary antibody against TNF-α (rabbit polyclonal IgG, 1: 200 dilution, cat. No. AP20373PU-N, Acris, Germany) and GFAP (rabbit polyclonal IgG at 1:200 dilution, cat. No. ab7260, Abcam, Cambridge, United Kingdom) at 4°C. Afterwards, the sections underwent incubation for 60 min with biotin-conjugated goat anti-rabbit IgG (Histofine kit, Nichirei Corporation, Japan) and for 30 min with streptavidin-peroxidase conjugate (Histofine kit, Nichirei Corporation, Japan). Diaminobenzidine (DAB) chromogen was added to inspect the streptavidin-biotin reaction. After counterstaining with hematoxylin, the slides were visualized by a pathologist using a light microscope.

The brown zones were measured as optical density (OD) in cornu ammonis 3 (CA3) and dentate gyrus of each rat for six rats per group employing an ImageJ software (National Institutes of Health, Bethesda, 150 MD, United States).

### 2.11 Statistical analysis

The statistical assessment of all data was executed by applying Graphpad Prism 5 (Graphpad Software, CA, United States). The Shapiro-Wilk approach was utilized to scrutinize the normality of the data. The numerical data were expressed as mean ± SEM. Two-way ANOVA followed by the Bonferroni *post hoc* test was implemented to analyze the data for the training session of the MWM test (escape latency data). Meanwhile, all other remaining results were statistically computed employing one-way ANOVA followed by Tukey’s *post hoc* test. The statistical significance threshold was set at p < 0.05.

## 3 Results

### 3.1 Effect of BISA on DOX-induced cognitive disorders

**FIGURE 1:**
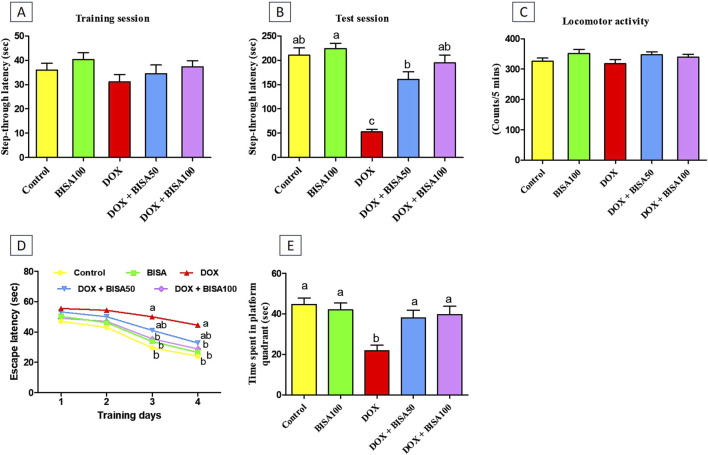
Impact of α-bisabolol on behavioral alterations caused by doxorubicin in rats. **(A)** Step-through latency (training session), **(B)** Step-through latency (test session), **(C)** Locomotor activity, **(D)** Morris water maze test (MWM, escape latency): 4 training days, **(E)** MWM (time spent in platform quadrant). All data were exhibited as mean ± SEM (n = 6). BISA50, BISA100: α-bisabolol was given by gavage at 50 and 100 mg/kg, DOX: doxorubicin. The findings for **(D)**, which represent the mean escape latency were statistically assessed by applying two-way ANOVA followed by Bonferroni *post hoc* test. Within each training day, different letters highlighted statistical variation (p < 0.05). Meanwhile, statistical investigation of other behavioral data was carried out employing the one-way ANOVA, then Tukey’s *post hoc* test. Bars designated with various lowercase letters are significantly different (p < 0.05).

No remarkable difference was recorded in the step-through latency among the tested groups throughout the training period of the passive avoidance experiment (Figure 1A). Throughout the test period, DOX administration significantly reduced (p < 0.05) the step-through latency by 75% relative to the control rats, indicating memory deterioration. BISA at 50 and 100 mg/kg/day along with DOX dose-dependently increased (p < 0.05) the step-through latency in comparison with those exposed to DOX alone (Figure 1B).

The locomotor activity was evaluated for all rats to preclude the influence of motor disorders on the outcomes of all executed neurobehavioral tests including the passive avoidance test. As displayed in Figure 1C, there were no statistical differences in the locomotor function among all groups.

The MWM test was performed to assess the learning and spatial memory of all rats. In the first 2 days of the training, all groups showed no significant difference in the mean escape latency time. DOX-treated rats exhibited lower capability to reach the hidden platform with a marked increase (p < 0.05) in escape latency time on the third and fourth days of the training session compared to the control group. In contrast, supplying DOX-treated rats with BISA, particularly at 100 mg/kg/day, resulted in significant reduction (p < 0.05) in the escape latency time compared to the DOX group (Figure 1D). On the fifth day (probe trial test), rats receiving DOX only exhibited a significant decrease (by 51%) (p < 0.05) in the time spent on the target platform quadrant in comparison with the controls. Conversely, the concurrent treatment with BISA (DOX + BISA50 and DOX + BISA100 groups) significantly increased (p < 0.05) the time spent on the target platform in a dose-dependent manner as compared to the DOX group (Figure 1E).

### 3.2 Effect of BISA on DOX-provoked hippocampal oxidative insult and on the mRNA levels of Nrf2 and HO-1 genes

DOX administration triggered a vigorous state of oxidative insult in the hippocampus manifested by a significant elevation (p < 0.05) in the content of lipid peroxidation marker (MDA) by 209% and a drastic decline (p < 0.05) of CAT, SOD and GSH levels (by 68%, 55%, and 60%, respectively) compared to the control group. Co-administration with BISA (at 50 and 100 mg/kg/day) reduced MDA levels and increased the CAT, SOD, and GSH levels in a dose-dependent manner. The DOX + BISA100 group showed better ameliorative effect on the redox status, where these oxidative insult biomarkers were restored to the control levels (Table 2).

**TABLE 2:** Effect of α-Bisabolol on DOX-induced oxidative insult indices in hippocampal tissues of experimental rats.

Experimental groups	MDA (nmole/g tissue)	CAT (U/g tissue)	SOD (U/g tissue)	GSH (mg/g tissue)
Control	11.78 ± 1.48^c^	2.15 ± 0.28^ab^	53.62 ± 4.34^ab^	18.36 ± 1.79^ab^
BISA	9.85 ± 0.86^c^	2.55 ± 0.33^a^	58.38 ± 3.97^a^	19.70 ± 2.23^a^
DOX	36.45 ± 4.20^a^	0.69 ± 0.10^c^	24.20 ± 3.18^c^	7.30 ± 0.95^c^
DOX + BISA50	23.18 ± 2.87^b^	1.25 ± 0.19^bc^	40.30 ± 3.87^b^	11.96 ± 1.18^bc^
DOX + BISA100	16.51 ± 1.55^bc^	1.88 ± 0.17^ab^	46.00 ± 3.55^ab^	15.76 ± 1.38^ab^

Values are delineated as the mean ± SEM (n = 6).

^a,b,c,d^different superscripts per column represent statistical significance (p < 0.05). BISA50, BISA100: α–Bisabolol was given by gavage at 50 and 100 mg/kg, DOX, doxorubicin; MDA, malondialdehyde; CAT, catalase; SOD, superoxide dismutase; GSH, reduced glutathione.

**FIGURE 2:**
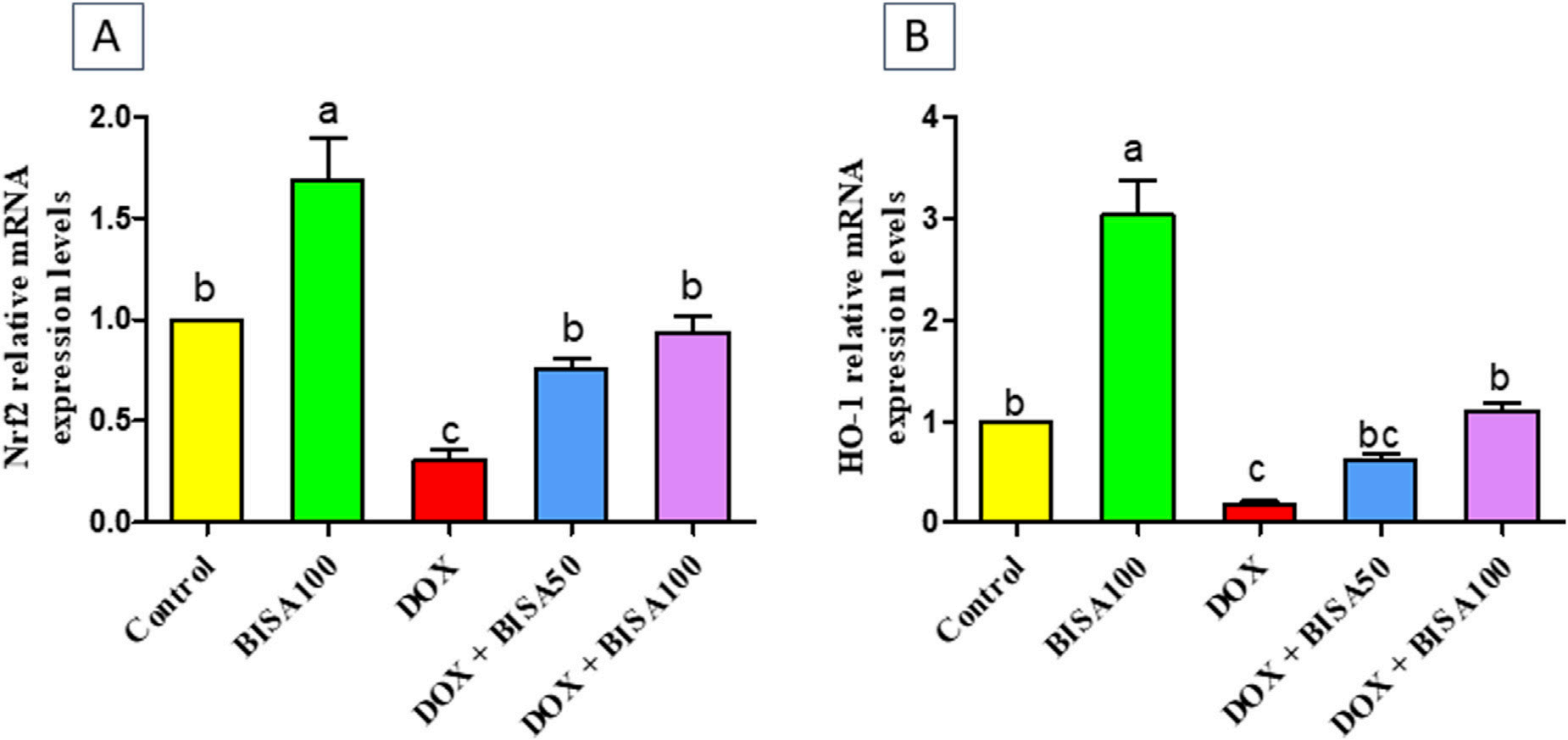
Impact of α-bisabolol on the transcription levels of Nrf2 **(A)** and HO-1 **(B)** in DOX-induced cognitive impairment in rats. Values are displayed as mean ± SEM (n = 6). The letters on every bar point out statistical significance between them (p < 0.05). BISA50, BISA100: α-bisabolol was administered orally at 50 and 100 mg/kg, DOX: doxorubicin was injected i. p. Once/week for 4 weeks, Nrf2: nuclear factor-erythroid 2-related factor-2, HO-1: heme oxygenase-1.

Furthermore, BISA caused a significant upregulation (p < 0.05) of Nrf2 gene and its antioxidant response element HO-1 in DOX + BISA50 and DOX + BISA100 groups compared to DOX group (Figures 2A,B).

### 3.3 Effect of BISA on DOX-induced histopathological alterations in the hippocampus

**FIGURE 3:**
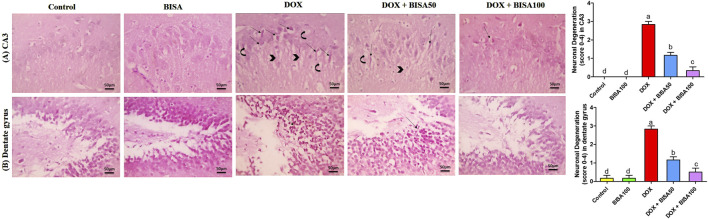
Microscopic photos of hippocampal slides from rats stained with H&E. **(A)** Hippocampal cornu ammonis 3 (CA3) sections, **(B)** hippocampal dentate gyrus sections. Slides from control and BISA100 (α-bisabolol oral administration at 100 mg/kg/day) groups exhibited no neuronal degeneration in both regions (CA3 and dentate gyrus). On the other hand, sections from the group treated with doxorubicin only (DOX group) revealed many degenerated neurons (thin arrows) with vacuolations (curved arrow) and neurofibrillar loss (arrowheads) in CA3 region and severe neuronal degeneration (thin black arrow) in dentate gyrus region. Tissues from DOX + BISA50 displayed fewer degenerated neurons (thin arrows) with vacuolations (curved arrow) and neurofibrillar loss (arrowhead) in CA3 region and showed decreased neuronal degeneration (thin black arrow)in dentate gyrus area. Hippocampal sections CA3 from DOX + BISA100 group exhibited few degenerated neurons (thin arrows), while denatte gyrus region from the same group revealed normal neurons. X400 bar 50 μm.

The control and BISA groups showed normal neuronal configurations in CA3 and dentate gyrus regions appeared as normal round intact neurons with normal lightly stained nucli, and no obvious degeneration was noticed. While the DOX-treated group displayed severe neuronal degeneration in these 2 regions observed as neuronal shrinkage, more eosinophilic cytoplasm and pyknotic nuclei besides vacuolations and neurofibrillar loss noticed only in CA3. Concurrent administration of BISA at 50 mg/kg/day with DOX reduced the neuronal degeneration in both regions. Moreover, the slides obtained from DOX + BISA100 group exhibited mild degeneration (<25%, scattered degenerated neurons) in CA3 and dentate gyrus areas. The quantitative data for these histological changes revealed statistical significance (P < 0.05) in the scores of degenerated neurons between DOX, DOX + BISA50, and DOX + BISA100 groups (Figures 3A,B).

**FIGURE 4:**
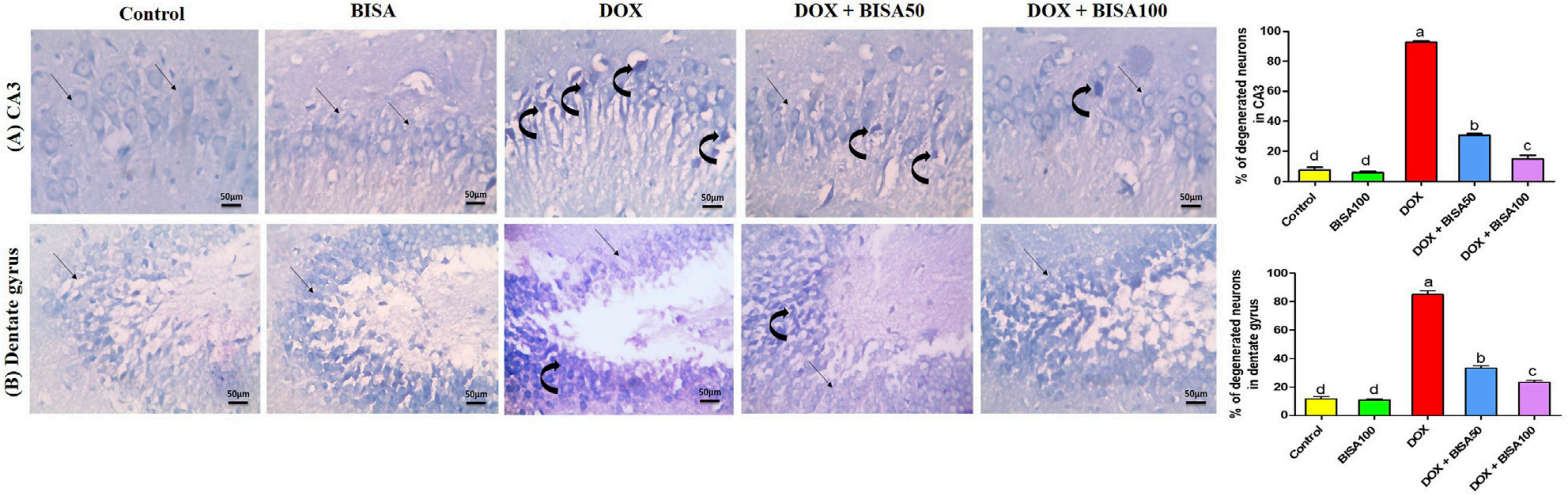
Photomicrographs of hippocampal sections stained with toluidine blue (X 400 bar 50 μm). **(A)** Hippocampal cornu ammonis 3 (CA3) sections, **(B)** hippocampal dentate gyrus sections. Control and BISA100 (α-bisabolol was gavaged orally at 100 mg/kg/day) groups showed normal blue-stained neurons (thin black arrow) in both regions of hippocampus (CA3 and dentate gyrus). On the contrary, sections from DOX-exposed rats showed many dark stained neurons (curved arrows) in the two regions. Slides from DOX + BISA50 presented fewer dark stained neurons (curved arrow) in the two hippocampal regions. DOX + BISA100 group showed many normal blue-stained neurons (thin black arrow) with few dark stained neurons (curved arrows) in CA3. The number of degenerated neurons was expressed as a percentage from 100 neurons in each area. Each bar labeled with various letters is significantly different (p < 0.05). Data are exhibited as mean ± SEM (n = 6).

These results were emphasized using toluidine blue stain. As shown in Figures 4A,B, CA3 and dentate gyrus regions from control and BISA100 groups revealed rounded neuronal build, normal cytoplasm, well-defined nuclei. In contrast, the two regions in the DOX-group exhibited prominent neuronal degeneration as indicated by loss normal rounded framework of normal neurons with darkened cytoplasm, hyperchromatic and pyknotic nuclei. DOX + BISA 50 group showed decrease in the number of degenerated neurons in CA3 and dentate gyrus regions and much more reduction was observed in DOX + BISA100. The percentage (%) of degenerated neurons in CA3 and dentate gyrus regions was shown in Figures 4A,B.

### 3.4 Impact of BISA on DOX-induced neuroinflammation and astrogliosis in the hippocampus

**FIGURE 5:**
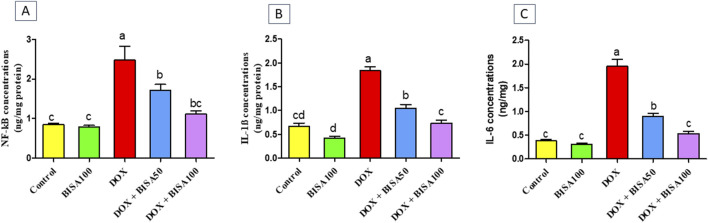
Effect of α-bisabolol on the concentrations of inflammatory markers detected by ELISA in hippocampus tissues of DOX-subjected rats. **(A)** NF-kB, **(B)** IL-1β, **(C)** IL-6. The results are shown as mean ± SEM (n = 6). Bars with different letters are substantially varied from one another (p < 0.05). BISA50, BISA100: α-bisabolol was offered at 50 and 100 mg/kg/day per os, DOX: doxorubicin was given i. p. One injection every week for 4 weeks, NF-kB: Nuclear factor kappa B, IL-1β: interleukin-1β, IL-6: interleukin-6.

The BISA100 group had no remarkable alterations in the hippocampal concentrations of the inflammatory markers including NF-kB (Figure 5A), IL-1β (Figure 5B), and IL-6 (Figure 5C). DOX administration provoked robust inflammatory reactions mirrored by the marked upsurge (p < 0.05) in the levels of these inflammatory indices in comparison with the control group. However, groups treated with DOX and BISA concurrently (DOX + BISA50 and DOX + BISA100 groups) showed a considerable reduction (p < 0.05) in the concentrations of these markers as compared to the rats receiving DOX alone. Moreover, no significant difference in the levels of NF-kB, IL-1β, and IL-6 was observed between the DOX + BISA100 group and the control group.

**FIGURE 6:**
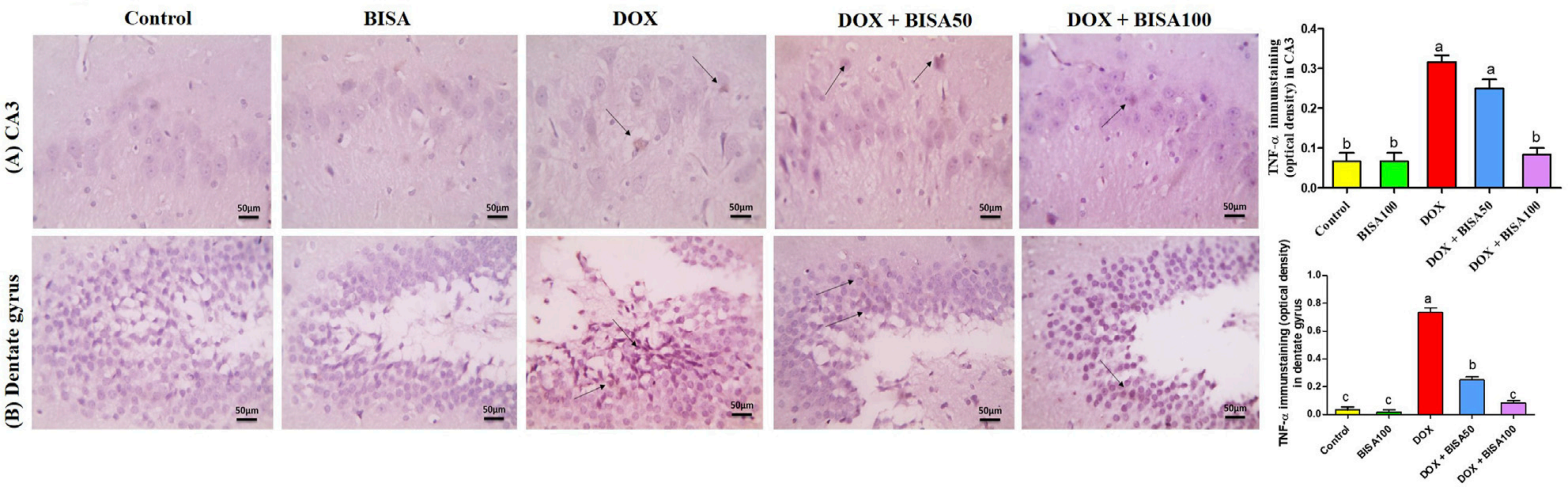
Histological images of immunostained hippocampal slides against tumor necrosis factor α (TNF-α) (X400 scale bar 50 μm). **(A)** Sections for hippocampal cornu ammonis 3 (CA3) region, **(B)** sections for hippocampal dentate gyrus region. Images from the control and BISA100-exposed rats revealed negative TNF-α expressions in CA3 and dentate gyrus. In contrast, DOX group showed positive brown staining for TNF-α in both examined areas of hippocampus (thin black arrow). The slides obtained from rats in DOX + BISA50 group displayed decrease in the positive brown staining for TNF-α in the two hippocampal areas (thin black arrow). DOX + BISA100 group presented mild positive brown TNF-α expression in the neurons in both screened regions (thin black arrow). The intensity of brown areas (TNF-α expression) in the experimental groups was exhibited as optical density (OD). Values were depicted as mean ± SEM (n = 6). There is a significance variation (p < 0.05) among bars that convey letters (p < 0.05).

The immunohistochemical investigation of the pro-inflammatory cytokine TNF-α revealed negative expression in the CA3 and dentate gyrus regions of the control and BISA100 groups. In contrast, a brown neuronal TNF-α expression was seen in these two regions of the rats treated with DOX only. Notably, concomitant administration of BISA at 50 mg/kg/day led to a reduction in the positive TNF-α staining in CA3 and dentate gyrus. Additionally, mild positive expression was detected in the tissues collected from rats treated with the higher dose of BISA (100 mg/kg/day) along with DOX. Quantitation of TNF-α staining was evaluated as optical density of the immunostained areas (Figures 6A,B).

**FIGURE 7:**
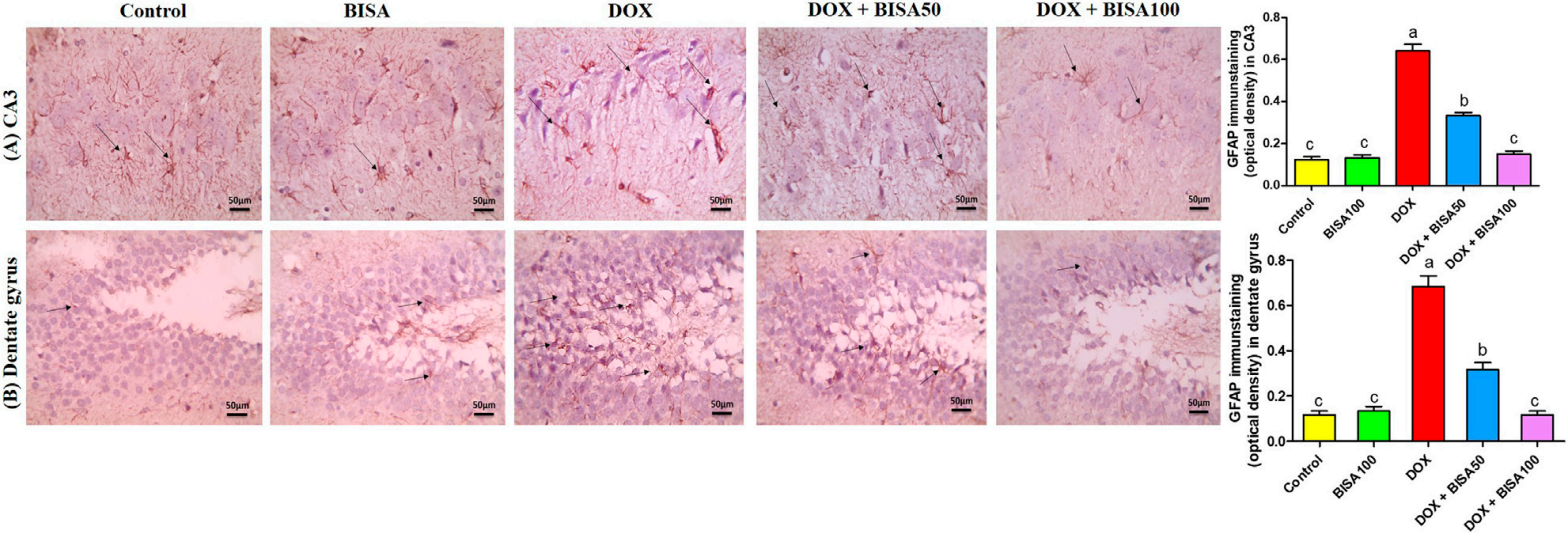
Immunohistochemical staining pictures for glial fibrillary acid protein (GFAP) in the hippocampus of experimental rats (X 400 scale bar 50 μm). **(A)** Images for cornu ammonis 3 (CA3) region, **(B)** images for dentate gyrus region. The sections from the control and BISA100 group exhibited few astrocytes in CA3 and dentate gyrus zones (thin black arrow). Conversely, the slides from DOX-treated rats revealed numerous highly branched brown stained astrocytes in the two inspected regions (thin black arrow). DOX + BISA50 showed fewer less branched astrocytes in both hippocampal regions (thin black arrow). DOX + BISA100 showed much fewer normal astrocytes in the two scrutinized hippocampal sections (thin black arrow). The brown areas (GFAP expression) were represented as optical density (OD). Data are reported as mean ± SEM (n = 6). Bars marked by different letters are significantly different (p < 0.05).

The immunohistochemical analysis of GFAP in the hippocampus displayed a few astrocytes in CA3 and dentate gyrus of the control and BISA100 groups. Multiple highly branched astrocytes were detected in the hippocampal sections of the DOX-treated rats. However, the slides from DOX + BISA50 group showed fewer less branched astrocytes in these two regions. Also, the DOX + BISA100 group showed very few astrocytes in these two regions. Quantitative assessment of immunohistochemcial staining for GFAP was represented as optical density (Figures 7A,B).

### 3.5 Impact of BISA on hippocampal apoptosis induced by DOX

**FIGURE 8:**
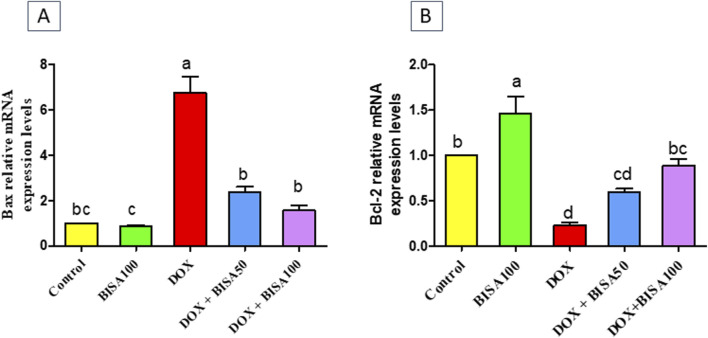
Effect of α-bisabolol on relative mRNA expression of apoptotic markers; **(A)** Bax and **(B)** Bcl-2 in the hippocampus of DOX-treated rats. Data are represented as mean ± SEM (n = 6). The lowercase letter on every bar clarify statistical difference among them (p < 0.05). BISA50, BISA100: α-Bisabolol was supplied by gavage at 50 and 100 mg/kg/day, DOX: doxorubicin was given i. p. Weekly for 4 weeks.

The results in Figure 8 showed that the mRNA level the pro-apoptotic gene (Bax) increased significantly (p < 0.05) in the hippocampus of DOX-treated rats when compared to the controls. On the other hand, a substantial downregulation (p < 0.05) in the mRNA level of the anti-apoptotic gene (Bcl-2) was detected in these DOX-exposed animals as compared to the controls. Whereas, DOX + BISA groups exhibited a significant suppression (p < 0.05) in Bax mRNA and an elevation (p < 0.05) in the Bcl-2 transcription level in comparison with DOX-group. The transcription levels of these apoptotic markers were completely reversed in rats receiving DOX plus BISA at 100 mg/kg/day.

### 3.6 Effect of BISA on synaptic plasticity of the hippocampus

**FIGURE 9:**
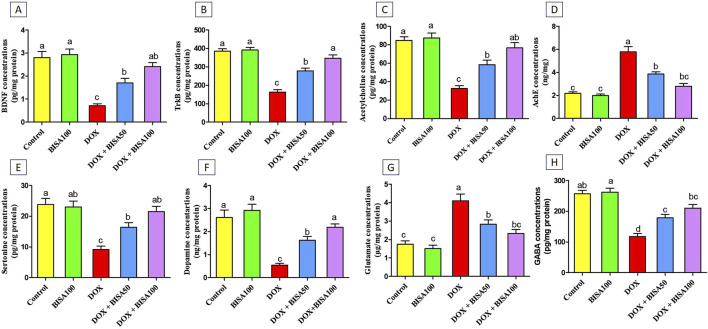
Effect of α-bisabolol on the protein expression of **(A)** BDNF, **(B)** TrKB, **(C)** Ach, **(D)** AchE, **(E)** 5-HT, **(F)** DA, **(G)** glutamate, **(H)** GABA in rat’s hippocampus in Dox-induced chemobrain. The findings are expressed as mean ± SEM (n = 6). Bars carrying various letters are substantially different (p < 0.05). BISA50, BISA100: α-bisabolol was introduced orally at 50 and 100 mg/kg/day, DOX: doxorubicin was injected i. p. Weekly for 4 weeks. BDNF: brain derived neurotropic factor, TrKB: tropomyosin receptor kinase B, ACh: acetylcholine, 5-HT: serotonin, DA: dopamine AChE: acetylcholinesterase, GABA: Gamma-aminobutyric acid.

To assess the effect of BISA on the synaptic plasticity, we evaluated the concentrations of BDNF and its receptor TrKB in the hippocampal tissues utilizing ELISA. Th The DOX treatment caused a significant reduction (p < 0.05) in the levels of BDNF and TrKB by 74% and 57%, respectively, compared to the control group. Conversely, BISA co-treatment at 50 and 100 mg/kg daily exhibited a noticeable rise (p < 0.05) in the concentrations of BDNF and TrKB in relation to rats receiving DOX solely. The entire restoration to the control concentrations of BDNF and TrKB was accomplished only with BISA at 100 mg/kg (Figures 9A,B).

Additionally, the concentrations of the neurotransmitters ACh, 5-HT, DA, and GABA were noteworthy decreased (p < 0.05) in the DOX group relative to the control one (Figures 9C,E,F,H). While, a substantial increase (p < 0.05) in AChE activity and glutamate level was recorded after DOX administration when compared to the control rats (Figures 9D,G). On the contrary, rats in the DOX + BISA50 and DOX + BISA100 groups displayed a marked elevation (p < 0.05) in ACh, 5-HT, DA, and GABA levels and a significant suppression (p < 0.05) in the activity of AChE and glutamate level in comparison with those in the DOX group. BISA at 100 mg/kg even returned their levels to near the control levels.

**FIGURE 10:**
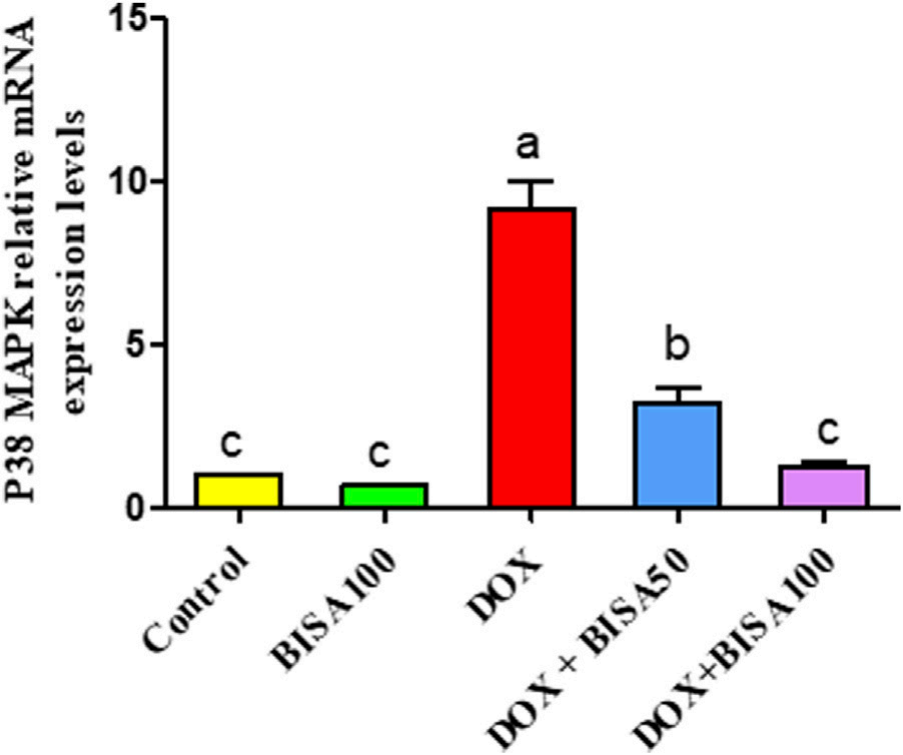
Influence of α-bisabolol on the relative mRNA expression of P38 MAPK in the hippocampus of rats exposed to DOX. The data are displayed as mean ± SEM (n = 6). Different letters on every bar point out statistical difference (p < 0.05). BISA50, BISA100: α-bisabolol was provided per os at 50 and 100 mg/kg/day, DOX: doxorubicin was given i. p. One injection per week for 28 days. P38 MAPK: P38 mitogen-activated protein kinase.

The qRT-PCR results demonstrated that the p38 MAPK mRNA level was remarkably higher (p < 0.05) in the hippocampus of rats treated with DOX only than the control rats. In contrast, both DOX + BISA groups markedly downregulated (p < 0.05) the mRNA expression of p38 MAPK compared to the DOX treatment alone. Furthermore, the transcription level of this gene returned to the normal in the DOX + BISA100 group (Figure 10).

### 3.7 Impact of BISA on the mRNA levels of SIRT-1 in the hippocampus

**FIGURE 11:**
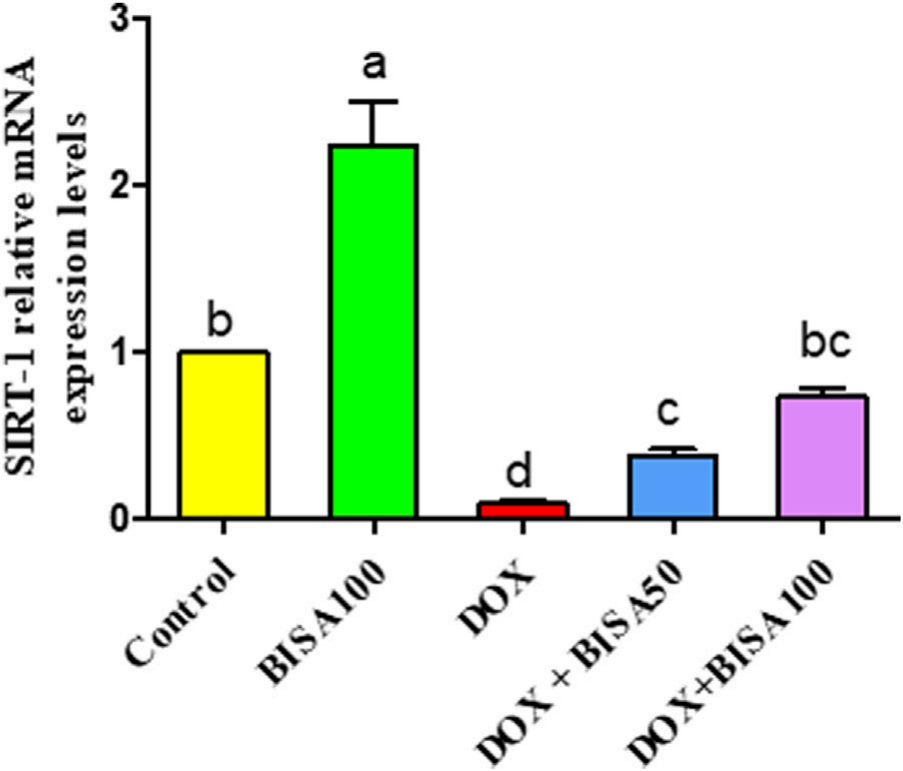
Impact of α-bisabolol on the relative mRNA expression of hippocampal SIRT-1 in DOX-induced cognitive impairment. Values are exhibited as mean ± SEM (n = 6). Bars with distinct lowercase letters are significantly different from one another (p < 0.05). BISA50, BISA100: α-bisabolol was gavaged at 50 and 100 mg/kg/day, DOX: doxorubicin was administered i. p. every week for 4 weeks. SIRT-1: sirtuin-1.

Figure 11 revealed a considerable downregulation (p < 0.05) in the transcription level of SIRT-1 in the DOX group in relation to control animals. Contrariwise, DOX + BISA50 and DOX + BISA100 groups showed a noticeable increase (p < 0.05) in the mRNA level of SIRT-1 gene counterweight to the DOX group. Notably, co-administration of BISA at 100 mg/kg with DOX revealed a better ameliorative action on SIRT-1 gene expression relative to the DOX + BISA50 group.

## 4 Discussion

The present study aimed to assess the potential neuroprotective effect of BISA against DOX-provoked chemofog. The results of our study revealed that BISA exhibited a palliative role against cognitive disturbances caused by DOX in rats, as shown by the enhancement of short-term memory and spatial learning, restoration of the hippocamapal histological architecture, alleviation of the oxidative stress and neuroinflammation, suppression of apoptosis, and reversion of the synaptic dysplasticity. The higher dose of BISA (100 mg/kg/day) displayed a more remarkable neuroprotective effect against DOX-induced chemofog than the lower dose (50 mg/kg/day). Our findings are consistent with our hypothesis that BISA has a palliative effect on DOX-induced chemofog via its antioxidant, anti-inflammatory, and anti-apoptotic properties, and its effect on the BDN/TrKB pathway.

In the present work, i. p. injection of DOX at 2 mg/kg/week for 4 weeks resulted in memory and learning disorders, as demonstrated by considerable shortening in the time of step-down latency of rats in the passive avoidance test, as well as prolongation in the time of the escape latency and reduction in the duration spent by the rats in the platform quadrant in the MWM test. Prior investigations showed that treatment of rats with DOX led to amnesia and deteriorations in their spatial cognitive abilities (Christie et al., 2012; Mounier et al., 2021; Wahdan et al., 2020). Considering that the hippocampus is primarily responsible for memory accession, storage, and recalling (El-Agamy et al., 2018), in keeping with the neurobehavioral manifestation, microscopic examination of hippocampal tissues collected from rats receiving DOX only presented severe neuronal degeneration. Meanwhile, the concurrent treatment with BISA alleviated the cognitive decline caused by DOX in both the behavioral and histoarchitectural conditions. In agreement with our results, Nazarinia et al. (2023) reported that oral BISA at 100 mg/kg/day for 10 days caused neurological protection and mitigated the memory impairment in rats experiencing seizures induced by pentylenetetrazole (PTZ).

The oxidative stress may be a major culprit in cognitive disorders caused by DOX. the quinone-containing compound (Joshi et al., 2010). Du et al. (2021) reported that the oxidative insult causes DOX-provoked cognitive dysfunction via direct and indirect mechanisms. DOX goes through a redox cycling process that involves the incorporation of a single electron to the quinone portion, producing a semi-quinone structure that is subsequently transformed back to the original quinone with the consequence of generating large quantity of reactive oxygen species (ROS) which harms both the quality of structure and performance of multiple crucial biological molecules, including lipids, proteins, and nucleic acids (Keeney et al., 2018). It has been reported that DOX-triggered oxidative insult causes elevation in lipid peroxidation and protein oxidation along with reduction in the concentrations of enzymatic and nonenzymatic antioxidants (Keeny et al., 2018). Previous studies revealed that DOX can evoke direct neuronal damage by enhancing the generation of ROS and the depolarization of mitochondrial neurons in hippocampal neurons (Du et al., 2021). Despite ROS is believed to perform an important role in triggering the neurotoxicity of DOX, the above mentioned theory is not reliable for introducing ROS into the CNS since DOX cannot penetrate the BBB (Bigotte et al., 1982). Hence, this suggests the probable involvement of an indirect process that does not include redox reactions in the brain. In parallel, Trachootham et al. (2008) demonstrated that free radicals produced from redox cycling enhance NF-κB, a redox-responsive transcriptional factor, with a subsequent stimulation of the expression of several inflammatory mediators, including TNF-α, IL-6, and IL-1β. Among these mediators of inflammation, TNF-α can pass the BBB and activate astrocytes, the main component of microglia, and are implicated in the synaptic transmission (Kimelberg and Nedergaard, 2010). Baune et al. (2012) revealed that astrocytes play a crucial role in the neuroinflammatory process as they react to inflammatory mediators’ insults inducing astrogliosis, a condition where the action of astrocytes is reversed to be deleterious and is associated with an excessive liberation of inflammatory signals. This results in a cycle of detrimental neurological inflammation that boosts the nitrosative and oxidative stresses and lowers synaptic plasticity. In this regard, inflammation has been considered to be another essential triggering factor, together with the oxidative insult, with cytokines representing an additional incorporating culprit in the progression of cognitive dysfunction caused by DOX (Aluise et al., 2010; El-Agamy et al., 2018).

In our research, the memory impairment caused by DOX was correlated with severe oxidative insults in the hippocampus, as represented by an upsurge in MDA concentration, a lipid peroxidation indicator, and a dramatic decline in SOD and CAT activities as well as the GSH level. In parallel, Joshi et al. (2005) reported that the neurological impacts of DOX are attributed to the heightened protein and lipid peroxidation alongside disorders in the anti-oxidant defense system. The imbalance in the redox status triggered by DOX in the present study can be illustrated by the remarkable downregulation of theNrf2 gene, a modulator of cytological defense against ROS, and its antioxidant response element HO-1 in the hippocamal tissues of DOX-treated rats. Our results are consistent with those of Liao et al. (2023), who reported that DOX administration in rats at 2.5 mg/kg for 7 injections caused a reduction in the expression of Nrf2 and in the mRNA level of HO-1 in hippocampal tissues. BISA attenuated this DOX-provoked oxidative stress. These findings agree with those of Nazarinia et al. (2023) who revealed that BISA alleviated the PTZ-provoked oxidative stress in the hippocampus of rats. Moreover, former researchers pointed out that BISA restored the activities of antioxidant enzymes such as SOD and CAT and suppressed the lipid peroxidation caused by DOX in renal, testicular, and cardiac tissues of rats (Arunacham et al., 2022; Arunacham et al., 2022; Meeran et al., 2023). Our results suggested that the palliative action of BISA against DOX-provoked chemofog is attributable to its antioxidant features. Previous reports revealed that BISA attenuated oxidative stress via scavenging ROS, suppressing their formation, and augmenting enzymatic antioxidant status (Meeran et al., 2020; Jeyakumar et al., 2022; Ramazani et al., 2022).

In addition, this research exhibited a remarkable inflammatory reaction in hippocampus after DOX injection as evidenced by a spike in the hippocampal levels of NF-κB, IL-6, and IL-1β as well as an increase in the immunoexpression of TNF-α and GFAP, the hallmark of astrogliosis. These findings are consistent with those of Elagmy et al. (2018) and Shaker et al. (2021). Conversely, BISA treatment alongside DOX attenuated this downstream inflammatory reaction. In the same vein, Javed et al. (2020) reported that BISA ameliorated the neurodegeneration in a ROT-provoked rat model of parkinsonism through abolishing the production of inflammatory cytokines such as IL-1β, IL-6, and TNF-α in the striatum. In this investigation, BISA’s anti-inflammatory effect in the hippocampus of DOX-exposed rats was associated with improvement in memory and learning abilities as observed from the outcomes of passive avoidance and MWM tests. In parallel, it has been proven that targeting neuroinflammation via suppressing several signaling encompassing NF-κB, NLR Family Pyrin Domain Containing 3 (NLRP3), and PI3K/protein kinase B (Akt), and their downstream pro-inflammatory cytokines rescues cognitive deficit associated with neurodegenerative disorders such as Alzheimer’s disease (Dempsey et al., 2017; Tyler and Tyler, 2023).

Earlier reports revealed that DOX-provoked redox imbalance led to mitochondrial impairment in the hippocampus. This malfunction might be a consequence of the emergence of mitochondrial permeability transition pores (Mptp), which results in bulging of the mitochondria and oozing of its liquids correlated with distortions in the mitochondria’s architecture (Javadov and Kuznetsov, 2013; Park et al., 2018). Bogner et al. (2010) reported that the disturbance in mitochondrial function after DOX treatment triggers the translocation of p53, a transcription factor that is pivotal for DNA repair and cytological proliferation/apoptosis, and enhances the splitting of Bax from Bcl-2 and B-cell lymphoma-extra-large (Bcl-xl), with the subsequent release of cytochrome C and eventually death of neurons through apoptosis. In parallel, our results showed that DOX administration led to neuronal apoptosis, witnessed by a marked rise in the mRNA level of Bax and a decline in the transcription of Bcl-2 in hippocampal tissues. BISA blocked DOX-induced apoptosis signaling, as indicated by the upregulation of Bcl-2 gene and downregulation of Bax gene. These findings are supported by former research which showed that BISA mitigated the apoptosis caused by ROT through boosting the expression of Bcl-2 and reducing cleaved caspase-3 and -9 levels in the striatum of ROT-treated rats (Javed et al., 2020).

The mitochondrial impairment, in addition to its role in apoptosis, may contribute to synaptic dysplasticity in the hippocampus (Park et al., 2018). In addition, the hippocampal synaptic plasticity is influenced by numerous neurotrophic agents, including BDNF, which is essential for stimulating neurogenesis and neuronal differentiation (Leal et al., 2015). According to Amidfar et al. (2020), BDNF plays a crucial role in cognitive function. Bathina and Das (2015) showed that the decreased BDNF level of the hippocampus is one of the mechanisms underlying the pathogenesis of neurodegenerative disorders. In this context, the present study revealed a reduction in the level of BDNF and its receptor. TrKB, a protein tyrosine kinase receptor that is abundantly expressed in the brain, was recognized as a receptor for BDNF and is vital for neurotransmission and neuron viability (Soppet et al., 1991; Minichiello, 2009). Our results are consistent with those of Alhowail et al. (2019) and Alhowail and Aldubayan (2021). BISA reversed this effect of DOX on BDNF and its receptor. To our best knowledge, we are the first researchers to report the effect of BISA on BDNF and TrKB. Other sesquiterpenoids such as β-caryophyllene have been proven to increase the levels of BDNF and TrkB in the hippocampus of rats exposed to aspartame-provoked cognitive dysfuntion (Rosa et al., 2023).

Teleanu et al. (2022) demonstrated that disruption in the homeostasis of neurotransmitters results in neurological disorders as cognitive impairment. Homeostasis in neurotransmitters can be affected by neurotransmitter loss, changes in redox state, oxidative insult, inflammation, metabolic disorders, alterations in synaptic protein concentrations, Ca^2+^ imbalance, and neuronal degeneration (Pradeepkiran and Reddy, 2020) This research showed that DOX-treated rats exhibited elevated levels of AChE and reduced ACh levels in the hippocampus. AChE is the enzyme to breakdown ACh. Hampel et al. (2018) reported that ACh in the hippocampus plays an essential role in preserving memory and other cognitive processes, and thus a decrease in ACh levels of the hippocampus leads to cognitive disorders. Elevated concentrations of AChE in brain tissues have been correlated with a lowering in ACh levels with a consequent decline in memory performance. Therefore, the concentration of AChE was recognized as an indicator for the severity of neurodegeneration triggered by dimentia (Santos et al., 2018; Mani, 2021). In addition, AChE has a potent effect on neuroinflammatory reaction, oxidative susceptibility, and apoptosis of neurons, incorporating markedly in the occurrence and progression of neurodegenerative conditions. A rise of AChE accounts for the reduction in the concentrations of ACh and IL-10, an anti-inflammatory cytokine; meanwhile, it is accompanied by elevated TNF-α, IL-1β, IL-18, and INF-γ levels (Walczak-Nowicka and Herbet, 2021). ACh hinders the liberation of cytokines in the parasympathetic anti-inflammatory signaling through which the nervous system regulates general inflammatory reactions (Borovikova et al., 2000). Thus, the neuroinflammatory effect of the elevated AChE may be due to the decrease in the ACh level. Our findings are consistent with those of Mani et al. (2022) who reported a rise in AChE levels in rats receiving DOX. In the present study, BISA blocked the effect of DOX on AChE and ACh in rats. Similarly, Jeyakumar et al. (2022) reported that BISA decreased AChE concentrations in Neuro-2a cells treated with β-amyloid (Aβ)_25–35._

Moreover, this investigation revealed that exposure to DOX led to a decline in the levels of 5-HT and DA. Kwatra et al. (2016) reported that the DOX treatment in rats decreased the hippocampal levels of these two monoamines that contribute significantly to cognitive function. 5-HT is a key regulator of synaptic plasticity in the hippocampus, and its deficiency impairs declarative memory and results in subpar performance in a novel object identification study (Fernandez et al., 2017). Schultz (2002) and Bäckman et al. (2006) revealed that DA exerts a vital function in cognitive processes and locomotor regulation. It was also found that changes in 5-HT and DA levels manifested in response to oxidative stress provoked by chemicals, genetic mutations that mimic oxidative insult and neurodegenerative disorders (Kita et al., 2003; Kraft et al., 2009; Ortiz et al., 2011). Du et al. (2021) demonstrated that the alterations in neurotransmitter concentrations (ACh, 5-HT, and DA) in the hippocampus result in disturbances in the conduction of nerve impulses, which eventually cause cognitive dysfunction. Our present work revealed that concomitant administration of BISA with DOX restored 5-HT and DA levels to their control values. The ameliorative action of BISA on 5-HT and DA may be due to its antioxidant characteristics and its capacity to mitigate oxidative stress.

We found that DOX increased hippocampal levels of glutamate. This action may be interpreted by the excessive release of TNF-α triggered by DOX, which in turn induces astrocytes to generate more glutamate neurotransmitters (Habbas et al., 2015). Haroon et al. (2017) reported that higher glutamate concentrations promote N-methyl-D-aspartate (NMDA) receptors with subsequent suppression of BDNF production and TrKB receptor, decreasing neurogenesis, proliferation of neurons, synaptic plasticity, and death of neurons. Notably, treatment with BISA blocked the DOX-provoked increase in glutamate level. This attenuating action of BISA on glutamate neurotransmitters could be explained by the inhibitory impact of BISA on TNF-α, which further resulted in hindering the synthesis of glutamate from astrocytes. To the best of the authors' knowledge, we are the first ones to report the effect of BISA on 5-HT, DA, and glutamate levels in the hippocampus.

Our study presented marked upregulation in the hippocampal transcription level of p38 MAPK, one of Mitogen-activated protein kinases (MAPKs), in DOX group. These outcomes are in accordance with those of Abd-El-Aal et al. (2022). ROS have been recognized to be the leading cause for p38 MAPK activation (Dröge, 2002). Kim and Choi, (2010) reported that MAPK signaling has a pivotal role in the pathogenesis of a variety of neurological illness. MAPKs, especially p38 MAPK, modulate the release of cytokines including TNF-α, IL-6, and IL-1β, pointing out their significant action in the process of inflammation provoked by oxidative stress (Son et al., 2011). Qiu et al. (2019) revealed that stimulation of p38 MAPK enhances inhibitor of nuclear factor kappa B (IκB) phosphorylation with subsequent moving of NF-κB to the nucleus and gene upregulation, leading to a cycle of persistent inflammatory reactions and damage to neurons. In addition, MAPKs have been reported to be implicated in apoptosis induction. Activated p38 MAPK can suppress certain proteins such as Bcl-xl, which naturally aid in ameliorating apoptosis, thereby exacerbate apoptosis (Yokota and Wang, 2016).

DOX-treated rats revealed a remarkable decrease in the mRNA level of SIRT-1, a key metabolic regulator that is essential for maintaining the function of mitochondria and prolonging the life span of the cells (Ng et al., 2015). The suppression of SIRT-1 transcription in rats receiving DOX only may be related to the enhancement of TNF-α expression. Guo et al. (2019) reported that TNF-α contributes to the downregulation of SIRT-1 expression. BISA reversed the upregulation of p38 MAPK gene and the downregulation of SIRT-1gene caused by DOX. Olmos et al. (2013) and Ren et al. (2019) revealed that SIRT-1, during oxidative insult, reduces the formation of ROS via boosting peroxisome proliferator-activated receptor-gamma coactivator alpha (PGC-1α) transcription and stimulates the enzymes responsible for detoxifying ROS. In addition, SIRT-1 depresses the expression of several inflammatory cytokines, including IL-1β and TNF-α through dampening NF-κB signaling (Ren et al., 2019). Furthermore, SIRT-1 is a major regulator of synaptic plasticity and cognitive performance via stimulating BDNF expression (Tang et al., 2020; Shen et al., 2018). We are the first, to the best of the authors knowledge, to report the modulating action of BISA on p38 MAPK and SIRT-1 expression.

The findings of this research provide the basis for the possible usage of BISA alongside DOX chemotherapy in cancer patients as a potential therapeutic strategy for ameliorating DOX-associated cognitive impairment. Future studies, including clinical trials, are crucial to evaluate the safety, long-term efficacy, and appropriate dosage regimen of BISA in cancer patients.

The present research addressed some limitations. First, our study focused only on studying the effects of BISA on memory, as one of the cognitive functions, in DOX-exposed rats. Thus, further study is warranted to assess the impact of BISA on other cognitive domains, such as attention, executive function, and problem-solving, to provide a more comprehensive understanding of its effects on DOX-induced cognitive impairment. Second, this research used a single time point for assessing the effect of BISA on DOX-induced oxidative stress, inflammation, neuronal degeneration and cognitive disorders. Further investigations using longitudinal assessment with multi-time points would give more complete picture of the BISA’s efficacy over time and the duration of its anti-inflammatory effect to explore the potential for relapse or rebound inflammation. Third, the study did not distinguish between generalized hippocampal oxidative stress and potential subregion-specific effects (e.g., CA1, CA3, dentate gyrus) as DOX and BISA may differentially affect specific hippocampal subregions.

## 5 Conclusion

This research highlighted the neuroprotective effect of BISA against DOX-induced cognitive disorders which was evidenced by enhancing the short-term memory, spatial memory, and learning abilities of rats in the behavioral tests. BISA exerted this effect through suppressing the oxidative insult of DOX, alleviating neuroinflammation, and apoptosis, in addition to improving synaptic plasticity via stimulating the hippocampal BDNF/TrKB signaling and modulating the neurotransmitters. The data of this study recommended an oral BISA at 100 mg/kg/day in rats to alleviate the neurological impairment caused by DOX. Future investigations are required to clarify the molecular mechanism by which BISA hinders chemotherapy-induced chemofog, in order to be regarded as a promising therapy in this respect.

## Data Availability

The original contributions presented in the study are included in the article, further inquiries can be directed to the corresponding author.

## Glossary

ACh: Acetylcholine
AChE: Acetylcholinestrase
Akt: protein kinase B
Bax: Bcl-2-associated X protein
Bcl-2: B-cell lymphoma 2
BBB: Blood brain barrier
BDNF: Brain derived neurotropic factor
BISA: a-Bisabolol
CA3: Cornu Ammonis 3,
CAT: Catalase
CICI: Chemotherapy-induced cognitive impairment
DA: Dopamine
DAB: Diaminobenzidine
DOX: Doxorubicin
ELISA: Enzyme-linked immunosorbent assay
GABA: Gamma-aminobutyric acid
GFAP: Glial fibrillary acid protein
GSH: Glutathione
HO-1: Heme oxygenase-1
IkB: Inhibitor of nuclear factor kappa B
5-HT: Serotonin (5-hydroxytryptamine)
IL-1B: Interleukin-1β
IL-6: Interleukin-6
MDA: Malondialdehyde
NF-kB: Nuclear factor kappa B
NLRP3: NLR Family Pyrin Domain Containing 3
NMDA: N-methyl-D-aspartate
Nrf2: Nuclear factor erythroid 2-related factor
OD: Optical density
P38 MAPK: P38 mitogen-activated protein kinase
P13K: phosphoinositide 3-kinases
QRT-PCR: Quantitative real time polymerase chain reaction
ROS: Reactive oxygen species
SIRT-1: Sirtuin-1
TNF-α: Tumor necrosis factor alpha
TrKB: Tropomyosin receptor kinase B
